# Evaluation of the performance of actions and outcomes in primary health care

**DOI:** 10.11606/S1518-8787.2017051006831

**Published:** 2017-09-11

**Authors:** Paula Vitali Miclos, Maria Cristina Marino Calvo, Claudia Flemming Colussi

**Affiliations:** I Programa de Pós-Graduação em Saúde Coletiva. Universidade Federal de Santa Catarina. Florianópolis, SC, Brasil; II Departamento de Saúde Pública. Universidade Federal de Santa Catarina. Florianópolis, SC, Brasil

**Keywords:** Primary Health Care, Basic Health Services, Health Evaluation, Health Services Evaluation, Efficiency, Organizational, Decision Making

## Abstract

**OBJECTIVE:**

The objective of this study has been to evaluate the performance of the primary care of Brazilian municipalities in relation to health actions and outcomes.

**METHODS:**

This is an evaluative, cross-sectional research, with a quantitative approach, aimed at the identification of the efficiency frontier of the primary care in health actions and outcomes in Brazilian municipalities. Secondary data have been collected from the *Programa Nacional de Melhoria do Acesso e da Qualidade da Atenção Básica* (National Program for Improving Access and Quality of Primary Care) and the Department of Informatics of the Brazilian Unified Health System, in 2012. The data envelopment analysis tool has been used for variable returns to scale with product orientation.

**RESULTS:**

Municipalities have been analyzed by population size, and small municipalities have presented a high percentage of inefficiency for both models.

**CONCLUSIONS:**

The analysis of efficiency has indicated the existence of a higher percentage of effective municipalities in the model of health actions than in the model of health outcomes.

## RESUMO

**OBJETIVO:**

Avaliar o desempenho da atenção básica dos municípios brasileiros quanto a ações e resultados em saúde.

**MÉTODOS:**

Pesquisa avaliativa, transversal, com abordagem quantitativa, para identificar a fronteira de eficiência da atenção básica em ações e resultados em saúde nos municípios brasileiros. Foi realizada coleta de dados secundários a partir do Programa Nacional de Melhoria do Acesso e da qualidade da Atenção Básica e do Departamento de Informática do Sistema Único de Saúde, no ano de 2012. Utilizou-se a ferramenta análise envoltória de dados para retornos variáveis de escala com orientação para produto.

**RESULTADOS:**

Os municípios foram analisados por porte populacional e verificou-se que para ambos os modelos, os municípios de pequeno porte apresentaram alto percentual de ineficiência.

**CONCLUSÕES:**

A análise da eficiência indicou a existência de um percentual maior de municípios eficientes no modelo de ações em saúde do que no modelo de resultados em saúde.

## INTRODUCTION

Since Brazil defined primary care (PC) as the main strategy to coordinate care in the service network and to achieve integrality in its various dimensions, efforts have been directed at expanding access and reducing inequalities in the provision of health actions and services, from the process of decentralization. Based on the principles of universality, accessibility, bonding, continuity of care, integrality of care, accountability, humanization, equity, and social participation, PC has promoted a model change in Brazil with the Family Health Strategy (FHS), focusing on the work of a multidisciplinary team and acting with a registered population^a^.

Despite the advances made in PC coverage since the origin of the Brazilian Unified Health System (SUS), the numerous challenges to achieve its principles affect its performance standards, which are different in each region of the country^1^.

Performance depends on the amount and types of resources used, and their relationship with the outcomes achieved. The municipal manager is responsible for the allocation of these resources in PC, and given the scarce and finite public resources, the need to discuss the efficiency and responsibility of managers in the use of these resources gains increasing importance, especially in a country with significant social and regional inequality^4^.

The efficiency of a health system can be defined as the relationship between the product of the health intervention and the resources used. It can also be established from an equation that considers the provision of care, with minimum waste, and the generation of a return corresponding to the volume of resources invested. In this sense, avoidable hospitalizations are an example of inefficiency, since they represent a waste of resources^b^.

In order to verify this relationship and to improve the efficiency of the PC, the federal government started a process of institutionalization of evaluation in PC in 2003^5^. Among the existing evaluation proposals, the *Programa Nacional de Melhoria do Acesso e da Qualidade da Atenção Básica* (PMAQ-AB – National Program for Improving Access and Quality of Primary Care) was created^c^, which induces the use of evaluation by managers and health teams and evaluates the performance of teams in relation to the quality standards and access agreed in a tripartite manner.

The evaluation is the judgment that is made on an intervention, or on any of its components, in order to assist in decision making^6^. Health assessment, and in this context PC evaluation, has the fundamental purpose of supporting decision-making processes, subsidizing the reorientation of actions and services, and measuring the impact of the actions implemented by services and programs on the health condition of the population^5^.

Considering the importance of PC evaluation and the importance of the efficient use of resources by municipal managers, this study has aimed to evaluate the performance of PC in Brazilian municipalities using the criterion of efficiency.

## METHODS

An evaluative transversal research with a quantitative approach was carried out to identify the frontiers of efficient practices in PC actions and outcomes in Brazilian municipalities, using data envelopment analysis.

The theoretical-logical model that guides this evaluative study is based on the theory of productive sectors, which considers the relation between analyzed inputs and products^7^. We understand that the selected inputs are fundamental for the development of the PC productive process and that there are combinations between them that provide a better cost-benefit relationship with the outcomes. Two different empirical models were elaborated: model 1, with emphasis on PC actions, and model 2, related to PC outcomes. We assume that both have a context with epidemiological, financial, socioeconomic, and political aspects, regulated by the legislation and guidelines of the public policies in force, which will guide municipal managers in their decision making regarding priorities in their municipality.

The next step after defining the theoretical-logical model was selecting the indicators, which was carried out in a consensus workshop, in the traditional committee format, with specialists (professors from two federal teaching institutions) in the areas of PC, Health Epidemiology, Health Assessment, and Data Envelopment Analysis. The Chart presents the inputs and products selected for each model.

**Box:** Inputs, products, and controls selected to identify the efficiency frontier in Model 1 (Health Actions) and Model 2 (Outcomes).

	Model 1 (Health Actions) Indicator	Source
Inputs	Number of Primary Care physicians	PMAQ-AB
	Number of Primary Care nurses	PMAQ-AB
	Number of basic health units with vaccination room	PMAQ-AB
	Number of basic health units with sonar or pinard	PMAQ-AB
Products	Number of home visits performed by Primary Care physician and nurse	PCIS
	Number of prenatal care appointments performed by Primary Care physician or nurse	PCIS
	Number of appointments or individual appointments performed by Primary Care nurse	PCIS
	Number of 3rd dose applied for tetra and pentavalent vaccines	IS-NIP
Controls	Number of Primary Care physicians per 1,000 inhabitants	PMAQ-AB/IBGE
	Number of Primary Care nurses per 1,000 inhabitants	PMAQ-AB/IBGE
	Percentage of basic health units with vaccination room	PMAQ-AB/IBGE
	Percentage of basic health units with devices – sonar or Pinard stethoscope	PMAQ-AB/IBGE

Model 2 (Outcomes)		

Insumos	Number of Primary Care physicians	PMAQ-AB:
	Number of Primary Care nurses	PMAQ-AB:
	Number of basic health units	PMAQ-AB:
Produtos	Percentage of live births with normal birth weight	SINASC
	Percentage of hospitalization from non-primary care sensitive conditions	HIS
Controles	Number of Primary Care physicians per 1,000 inhabitants	PMAQ-AB/IBGE
	Number of Primary Care nurses per 1,000 inhabitants	PMAQ-AB/IBGE
	Number of Basic Health Units per 1,000 inhabitants	PMAQ-AB/IBGE

PCIS: Primary care information system; IS-NIP: Information system of the national immunization program; PMAQ-AB: National program for improving access and quality of the primary care; IBGE: Brazilian institute of geography and statistics; SINASC: Live birth information system; HIS: Hospital information system

Some products were included to control the corresponding inputs (controls). Their values seek to minimize the effect of productivity logic on the establishment of the frontier, thus avoiding that insufficient inputs generate efficiency.

A municipality with low medical coverage could be considered as efficient given its relationship between inputs and products. However, the control product for this case, rate of physicians per inhabitant, will be low and will prevent the municipality from being considered as efficient if the number of physicians is far below the recommended level. For municipalities with up to 5,000 inhabitants, the controls were not used in the model, since many municipalities in this stratum have high rates of physicians and nurses per inhabitant.

With the definition of the indicators, 2012 was established as the reference year for data collection because of the existence of the published data of the PMAQ. The universe of the research considered all Brazilian municipalities as Decision Making Units (DMU), using 2012 as base year for all data, amounting to 5,565 municipalities. The secondary data were obtained from: a) the database of the unit census, carried out in the PMAQ-AB; b) the *Sistema de Informação da Atenção Básica* (PCIS – Primary Care Information System), the *Sistema de Informação do Programa Nacional de Imunização* (IS-NIP – Information System of the National Immunization Program), and the *Sistema de Informações sobre Nascidos Vivos* (SINASC – Live Birth Information System), of the Department of Informatics of the Brazilian Unified Health System (DATASUS); and c) the population base of the Brazilian Institute of Geography and Statistics (IBGE).

To verify the consistency of the collected data, they were analyzed using historical series or, in the case of the PCIS, analyzed monthly. When errors were suspected, they were compared with records in other systems or the sample element was eliminated from the study.

The performance analysis assumes that there are homogeneous groups of units so that results can be compared. To this end, Brazilian municipalities were stratified and analyzed using population size as a criterion to establish homogeneous groups. Six dimensions were considered: size 1, with municipalities of up to 5,000 inhabitants; size 2, with municipalities from 5,001 to 10,000 inhabitants; size 3, with municipalities from 10,001 to 20,000 inhabitants; size 4, with municipalities from 20,001 to 50,000 inhabitants; size 5, with municipalities from 50,001 to 100,000 inhabitants; and size 6, with municipalities with more than 100,000 inhabitants. Larger municipalities (size 6) were excluded because of their small number, since the literature indicates that the amount of DMU should be at least three times greater than or equal to the sum of the materials and inputs used for variable returns to scale (DEA-BCC)^7^. All data were submitted to exploratory statistical analysis.

For both models, municipalities that were not part of the 2012 PMAQ (n = 22) and municipalities with PC coverage of less than 80% (n = 1,436) were excluded. Subsequently, we analyzed the variables of “number of physicians”, “number of nurses”, and “number of teams”, from the PMAQ-AB database, which resulted in the exclusion of another 802 municipalities whose data did not show consistency or completeness.

Specifically for model 1, municipalities with zero inputs (n = 178) were also excluded and, to homogenize the data, we analyzed the distributions of the rates of home visits by PC physicians and nurses, prenatal care, physician per inhabitant, nurse per inhabitant, and nursing appointments, without distinction of size, and the municipalities whose values were above the 95th percentile and below the 5th percentile (n = 1,102) were eliminated. In model 2, to homogenize the sample, we calculated the 5th and 95th percentiles of the rate of products of “physician per inhabitant”, “nurse per inhabitant”, and “basic health unit per inhabitant” and the municipalities with values below the 5th percentile and above the 95th percentile (700 municipalities) were eliminated.

Approximately 2,011 municipalities remained in model 1 and 2,595 municipalities remained in model 2; their distribution by population size and Brazilian region can be found in Table 1.

**Table 1:** Distribution of selected municipalities for model 1 (health actions) and model 2 (outcomes) according to population size and region. Brazil, 2015.

Size (1,000 inhabitants)	Region	Municipalities	Model 1		Model 2	
		
		Total	n	%	n	%
1 (up to 5)	Midwest	144	57	39.6	85	59.0
	North	84	27	32.1	46	54.8
	Northeast	240	95	39.6	118	49.2
	South	435	140	32.2	209	48.0
	Southeast	395	100	25.3	168	42.5
	Total	1,298	419	32.3	626	48.2
2 (5 ┤ 10)	Midwest	103	50	48.5	62	60.2
	North	83	19	22.9	19	22.9
	Northeast	366	191	52.2	237	64.8
	South	268	81	30.2	108	40.3
	Southeast	390	135	34.6	186	47.7
	Total	1,210	476	39.3	612	50.6
3 (10 ┤ 20)	Midwest	108	45	41.7	54	50.0
	North	106	23	21.7	30	28.3
	Northeast	587	335	57.1	415	70.7
	South	232	99	42.7	128	55.2
	Southeast	355	146	41.1	199	56.1
	Total	1,388	648	46.7	826	59.5
4 (20 ┤ 50)	Midwest	76	30	39.5	32	42.1
	North	113	17	15.0	19	16.8
	Northeast	427	224	52.5	261	61.1
	South	152	41	27.0	47	30.9
	Southeast	287	90	31.4	105	36.6
	Total	1,055	402	38.1	464	44.0
5 (50 ┤ 100)	Midwest	17	3	17.6	2	11.8
	North	39	3	7.7	4	10.3
	Northeast	115	37	32.2	38	33.0
	South	53	8	15.1	8	15.1
	Southeast	102	15	14.7	15	14.7
	Total	326	66	20.2	67	20.6

A positive correlation was found between inputs and products of the proposed models (p < 0.05).

To analyze the data under the criterion of efficiency, the DEA-BCC model with product orientation was chosen. This choice is justified by the hypothesis that resources in the health sector are scarce and that the municipal manager must make choices in order to offer the maximum number of health actions to the population with the resources available. We used the Max-DEA software, which is free and available at http://www.maxdea.cn.

## RESULTS

Table 2 shows the average, standard deviation, minimum, and maximum values of the variables used in the study, according to population size. We can observe in models 1 and 2 that there is still a great heterogeneity among the data of the municipalities of the same population size despite the exclusion criteria to homogenize the sample.

**Table 2:** Descriptive statistics of inputs (M) and products (P) that comprised the model of health actions (model 1) and the model of outcomes (model 2). Brazil, 2012.

Size		Variable of Model 1												Variable of Model 2							
	
		I1	I2	I3	I4	P1	P2	P3	P4	P5	P6	P7	P8	I1	I2	I3	P1	P2	P3	P4	P5
1	Average	1.5	1.5	1.2	1.3	483.9	243.1	0.5	0.5	1.836.4	40.3	83.3	88.5	1.5	1.5	1.5	0.4	0.5	0.4	67.8	92.1
	SD	0.6	0.6	0.4	0.5	333.4	134.2	0.1	0.1	1.450.4	17.6	25.9	22.6	0.6	0.6	0.6	0.1	0.1	0.1	12.8	5.2
	Min.	1	1	1	1	52	17	0.2	0.2	126	6	20	20	1	1	1	0.2	0.2	0.2	17.1	69.2
	Max.	4	3	3	4	1.745	661	0.9	0.8	7.279	117	100	100	4	3	3	0.9	0.8	0.8	100	100
2	Average	3.2	3.2	1.9	2.4	951.4	530.9	0.4	0.4	4.478.5	94.1	62.7	77.8	3.2	3.1	3.2	0.4	0.4	0.4	65.4	92.2
	SD	1.1	1	0.9	1	607.7	243.8	0.1	0.1	2.780.7	31	28.6	25.6	1.1	1	1	0.1	0.1	0.1	12.6	3.4
	Min.	2	2	1	1	134	33	0.2	0.2	431	29	10	10	2	2	2	0.2	0.2	0.2	20.6	75.4
	Max.	7	7	5	7	3.034	1.131	0.9	0.8	13.793	246	100	100	7	7	7	0.9	0.8	0.8	100	100
3	Average	6.1	6	3.6	4.6	1.601.4	1.086.5	0.4	0.4	9.905.1	199.1	61.9	78.8	5.9	5.9	5.9	0.4	0.4	0.4	62.2	92.5
	SD	2.2	1.9	1.8	1.7	1.063.3	511.3	0.1	0.1	5.465.2	65.2	28.1	22.7	2.1	1.9	1.9	0.1	0.1	0.1	13.5	2.4
	Min.	3	3	1	1	268	56	0.2	0.2	762	68	8.3	14.3	3	3	3	0.2	0.2	0.2	8.3	80.4
	Max.	17	14	10	10	6.264	2.554	0.9	0.8	27.690	448	100	100	17	14	14	0.9	0.8	0.8	95.3	98.2
4	Average	10.9	10.8	7	8.4	2.786	2.219.3	0.4	0.4	20.224.2	419.4	67.7	81.1	10.8	10.7	10.6	0.4	0.4	0.4	62.8	92.3
	SD	3.7	3.4	3.1	2.8	1.931.4	1.058.1	0.1	0.1	10.725.5	142.4	25.6	19.1	3.7	3.4	3.4	0.1	0.1	0.1	11.9	1.9
	Min.	5	6	1	2	630	117	0.2	0.2	1.420	166	5.3	12.5	5	5	5	0.2	0.2	0.2	13.5	86.4
	Max.	32	25	19	19	15.385	6.030	0.9	0.7	64.642	954	100	100	32	25	25	0.9	0.7	0.8	85.7	98.5
5	Average	22	21.4	14.2	16.9	5.174	4.877.4	0.3	0.3	43.195.1	958.7	70.5	83.2	21.6	21.1	20.7	0.3	0.3	0.3	66.2	92.1
	SD	5.1	4.5	4.8	4.6	2.528.3	1.989.8	0.1	0.1	18.517.8	258.4	23	17.1	5.1	4.6	5.4	0.1	0.1	0.1	10.8	1.5
	Min.	14	14	4	5	1.886	931	0.2	0.2	10.634	447	23.3	33.3	14	13	14	0.2	0.2	0.2	36.1	89.5
	Max.	35	32	26	28	13.523	10.097	0.5	0.5	97.744	1.731	100	100	35	32	43	0.5	0.5	0.6	84	95.9

SD: standard deviation; Min.: Minimum; Max.: Maximum; BHU: basic health unit

Model 1: M1 – Number of physicians; M2 – Number of nurses; M3 – Number of BHU with vaccination room; M4 – Number of BHU with sonar/pinard; P1 – Number of home visits; P2 – Number of prenatal care appointments; P3 – Number of physicians per inh.*1,000; P4 – Number of nurses per inh.*1,000; P5 – Number of nursing appointments; P6 – Number of 3rd dose applied for tetra and pentavalent vaccines; P7 – Percentage of BHU with vaccination room; P8 – Percentage of BHU with sonar/pinard.

Model 2: M1 – Number of physicians; M2 – Number of nurses; M3 – Number of BHU; P1 – Number of physicians per inh.*1,000; P2 – Number of nurses per inh.*1,000; P3 – Number of BHU per inh.*1,000; P4 – Number of hospitalizations for other causes; P5 – Percentage of live births with normal birth weight.

For each population size, an efficiency frontier was estimated. The municipalities that were below the frontier are considered as inefficient, that is, given the proposed models, they should perform more health actions than they are offering (model 1) or obtain more health outcomes than they are achieving (model 2).

Municipalities at the efficiency frontier are the municipalities of reference. In all sizes, we identified the municipalities that were reference only for themselves and those that were reference for more than one DMU. A municipality being an efficient reference only for itself suggests a combination of inputs and products so peculiar that perhaps it cannot be adopted anywhere else; in addition, the more an efficient municipality is a reference for other municipalities, the stronger is the indication that its combination of inputs and products can be reproduced and adopted as an example by other municipalities.

Table 3 illustrates the percentage of municipalities that are inefficient, efficient reference only for themselves, and efficient reference for more than one DMU, according to population size. We can observe that, in both models, size 1 showed a higher concentration (above 90% of the sample) of inefficient municipalities, while this value was lower in other models.

**Table 3:** Distribution of municipalities into inefficient, efficient for itself, and efficient for more than one DMU in the model of health actions (model 1) and the model of outcomes (model 2), according to population size. Brazil, 2012.

Population size	Inefficient		Efficient for itself		Efficient for more than 1 DMU	
		
	n	%	n	%	n	%
Model 1	1,284	63.8	362	18.0	365	18.2
Size 1	378	90.2	16	3.8	25	6.0
Size 2	282	59.2	93	19.5	101	21.2
Size 3	384	59.3	142	21.9	122	18.8
Size 4	221	55.0	84	20.9	97	24.1
Size 5	19	28.8	27	40.9	20	30.3
Model 2	2,325	89.6	110	4.2	160	6.2
Size 1	613	97.9	3	0.5	10	1.6
Size 2	540	88.2	23	3.8	49	8.0
Size 3	729	88.3	38	4.6	59	7.1
Size 4	403	86.9	29	6.3	32	6.9
Size 5	40	59.7	17	25.4	10	14.9

DMU: Decision making units

Size 1: up to 5,000 inhabitants; Size 2: 5,000 ┤ 10,000 inhabitants; Size 3: 10,000 ┤ 20,000 inhabitants; Size 4: 20,000 ┤ 50,000 inhabitants; Size 5: 50,000 ┤ 100,000 inhabitants.

Table 4 considers the 1,880 municipalities present in both models. We can observe that no municipality of size 1 was efficient and 88.1% of them were inefficient in both models. The efficient municipalities in the two models represent 7.7%, with 1% being efficient reference only for themselves and 2.9% for several municipalities in both models.

**Table 4:** Number and percentage of municipalities according to classification of efficiency and population size. Brazil, 2012.

Efficiency	Porte do município n (%)					

	1	2	3	4	5	Total
Inefficient in the two models	332 (88.1)	238 (56.4)	362 (58.0)	211 (53.3)	13 (21.3)	1,156 (61.5)
Inefficient in one model and efficient for several municipalities in the other	30 (8.0)	81 (19.2)	90 (14.4)	65 (16.4)	11 (18.0)	277 (14.7)
Efficient only for itself in only one of the models	15 (4.0)	72 (17.1)	120 (19.2)	79 (19.9)	16 (26.2)	302 (16.1)
Efficient for itself in the two models	-	3 (0.7)	5 (0.8)	4 (1.0)	7 (11.5)	19 (1.0)
Efficient for itself in one model and for several municipalities in the other	-	14 (3.3)	27 (4.3)	19 (4.8)	12 (19.7)	72 (3.8)
Efficient for several municipalities in the two models	-	14 (3.3)	20 (3.2)	18 (4.5)	2 (3.3)	54 (2.9)

Total	377 (100)	422 (100)	624 (100)	396 (100)	61 (100)	1,880 (100)

Size 1: up to 5,000 inhabitants; Size 2: 5,000 ┤ 10,000 inhabitants; Size 3: 10,000 ┤ 20,000 inhabitants; Size 4: 20,000 ┤ 50,000 inhabitants; Size 5: 50,000 ┤ 100,000 inhabitants.

The DEA-BCC model with product orientation indicates the equiproportional expansion of product(s) so that the municipality reaches efficiency. In model 1, the result indicates that home visits of PC physicians and nurses should be increased by 62%, which means almost two million appointments. Prenatal care appointments must increase 22%, which means approximately 492,000 more appointments. For nursing appointments, the number reaches more than seven million appointments (35% more) and when we observe the tetra/pentavalent vaccination coverage for children under one year of age, the value that must be increased represents 12%, which is equivalent to more than 50 thousand vaccines. In model 2, it is necessary to increase 2% of live births with normal birth weight, representing almost 6,000 births, and almost 33,000 hospitalizations for primary care sensitive conditions could be avoided.

## DISCUSSION

The performance in the health area remains an important parameter for managers to conduct their actions and guarantee the access and quality of health services to the population.

This study identified, using the criterion of efficiency, the performance of Brazilian municipalities in PC, based on health actions performed at this level of care and their outcomes. The municipalities were analyzed by population size and, for both models, the high percentage of inefficiency of small municipalities stood out among the other population sizes. When comparing the models, the model that emphasizes health actions (model 1) had a higher percentage of efficient municipalities.

The inputs used – financial and professional equipment and materials – decrease as population increases, and products suffer variation with size only for municipalities with more than 100,000 inhabitants^8^. Thus, economies of scale may justify the greater inefficiency of small municipalities. However, the care model can also influence productivity, and performance can be improved with the adoption of programs with financial incentives and the increase in the number of procedures offered by other professionals^9^.

Small municipalities have their own characteristics. We can highlight the turnover of professionals that integrate health teams, FHS units covering rural areas with low population density, and particular restrictions on working conditions. In addition, we can mention the economic inefficiency by nature, indicating the difficulty of collecting taxes to pay the expenses related to the provision of health services to the population^10^.

The possibility of efficiency in actions without consequent efficiency in outcomes has already been verified in Santa Catarina, Southern Brazil, in a study of actions and outcomes related to systemic arterial hypertension (SAH) in municipalities with up to 10,000 inhabitants. The number of municipalities that are efficient in the production of appointments and examinations was more than double of the municipalities efficient in avoiding hospitalizations from hypertension^13^.

In addition to the differences between the Brazilian regions, we can observe that the evaluations of the services offered by the PC are also influenced by population size, since small and medium-sized municipalities show a service organization and health reality different from that found in the large urban centers^14^. The context of large urban centers is a challenge for the reorganization of the PC model using the FHS, since they have a large and complex network of health services offered by the traditional model and private services. In these municipalities, poverty, social inequalities, violence, crime, unemployment, and an uncoordinated and poorly distributed health care network stand out, among other characteristics. Such considerations highlight the problems of these places and require not only public health policies, but also the collaboration of public policies that are related to urban development^15^.

The criterion of efficiency alone does not evaluate access and quality, but it indicates the practices of the input-product relationship that can be a reference for the optimization of the limited resources in PC. In this criterion, higher efficiency percentages were observed for health actions than for outcomes. In addition, municipalities with 50,000 to 100,000 inhabitants had the highest efficient percentage for actions and outcomes. This result may be associated with economy of scale. However, when other criteria such as access, quality, effectiveness and, performance of the health system are evaluated, other population sizes have better outcomes^14^.

In fact, when analyzing the references for inefficient municipalities to reach the efficiency of model 1, we highlight two actions that need to increase more than 30% so that municipalities can become efficient: home visit by physicians and nurses and nursing appointments. These actions are characteristic of the FHS-based care model, which proposes a greater link between the professionals of the primary health unit and the community and the decentralization of activities from the physician, highlighting the role of a multidisciplinary team.

In some cases, home visits may show the difficulties of some professionals in joining this activity. Home visits are rarely performed by a physician^16^, and is usually mediated by nurses and community health agents to define the criteria for visits.

The role of nurses in nursing appointments in PC is an important task for the change of the care model focused on the physician. Their inclusion in the multidisciplinary team can increase the access of the population to health appointments^17^.

For the objectives of model 2, we highlight the relative reduction of hospitalizations for primary care sensitive conditions, which would reflect the access, quality, coverage, and resolutiveness of the PC. The reduction of hospitalizations for primary care sensitive conditions indicates the strengthening of the PC with consequent reduction of costs from avoidable hospitalizations^12^ ^,^ ^18^.

The results of this study indicated that being efficient in actions does not guarantee the efficiency in outcomes. It is known that factors peculiar to local health systems can influence the resolution of certain actions. Municipalities with better practices, which are a reference to others, allow the establishment of possible goals in organizational improvement^19^, but local factors should be considered for adjustments to the reality of each municipality.

The process of decentralization in the SUS placed the municipality as responsible for the supply of health actions and services in PC, thus allowing a diversity of means and results of implementation of the FHS^20^. One of the concerns of SUS managers is the monitoring of health actions related to PC, as well as the outcomes of these actions on the health of the population served^21^.

Health actions in PC, guaranteed by specific guidelines and legislation, are prioritized locally by the municipal manager. This management defines the great diversity of care models and priorities in the municipalities, which have singular characteristics to be considered.

The evaluation of the performance of these health actions, as well as their outcomes, is necessary when the reduction of inequalities of PC access is proposed from the reorganization of the care model using the FHS. Among the various evaluation criteria, efficiency helps municipal managers in making decisions regarding the best allocation of resources. Moreover, given a global context with increasingly scarce health resources, the optimization of the inputs used to provide the necessary services is crucial to meet the population’s demand.

## Footnotes

a Ministério da Saúde (BR). Portaria Nº 2.488, de 21 de outubro de 2011. Aprova a Política Nacional de Atenção Básica, estabelecendo a revisão de diretrizes e normas para a organização da Atenção Básica, para a Estratégia Saúde da Família (ESF) e o Programa de Agentes Comunitários de Saúde (PAC). *Diário Oficial União*. 24 out 2011 [cited 2017 Apr 13];Seção 1:48. Available from: http://bvsms.saude.gov.br/bvs/saudelegis/gm/2011/prt2488_21_10_2011.html

b Viacava F, coordenador. PROADESS: avaliação de desempenho do sistema de saúde brasileiro: indicadores para monitoramento: relatório final. Rio de Janeiro: Fiocruz, ICICT; 2011 [cited 2017 Apr 13]. Available from: http://www.proadess.icict.fiocruz.br/SGDP-RELATORIO_FINAL%20_com_sumario_atualizadorev%202014.pdf

c Ministério da Saúde (BR). Portaria Nº 1.654, de 19 de julho de 2011. Institui, no âmbito do Sistema Único de Saúde, o Programa Nacional de Melhoria do Acesso e da Qualidade da Atenção Básica (PMAQ-AB) e o Incentivo Financeiro do PMAQ-AB, denominado Componente de Qualidade do Piso de Atenção Básica Variável - PAB Variável. Brasília (DF); 2011 [cited 2017 Apr 13]. Available from: http://bvsms.saude.gov.br/bvs/saudelegis/gm/2011/prt1654_19_07_2011.html
